# Safranal acts as a neurorestorative agent in rats with cerebral ischemic stroke via upregulating SIRT1

**DOI:** 10.3892/etm.2023.12358

**Published:** 2023-12-19

**Authors:** Fei He, Chunmian Chen, Yangyang Wang, Shuen Wang, Shuangyan Lyu, Junqiang Jiao, Guoyong Huang, Jiangshun Yang

**Affiliations:** 1 Department of Rehabilitation Medicine, Wenzhou Seventh People's Hospital, Wenzhou, Zhejiang 325006, P.R. China; 2 Key Laboratory of Neuropsychiatric Endocrinology, Wenzhou Seventh People's Hospital, Wenzhou, Zhejiang 325006, P.R. China

**Keywords:** ischemic stroke, safranal, SIRT1, neuron survival, neurogenesis, angiogenesis

## Abstract

Safranal is an active ingredient of saffron (*Crocus sativus L*.). Its neuroprotective role in ischemic stroke (IS) through reducing oxidative stress damage has been widely reported. However, the neurorestorative mechanisms of safranal are still in the preliminary stage of exploration. the present study is aimed to discuss the effects of safranal on the recovery of neural function after IS. A middle cerebral artery occlusion/reperfusion (MCAO/R) rat model and an oxygen-glucose deprivation/reoxygenation (OGD/R) model in rat brain microvascular endothelial cells (RBMEC) were established to explore the effects of safranal on IS *in vivo* and *in vitro*. It was found that safranal dramatically reduced infarct size and Nissl's body loss in rats subjected to MCAO/R. Safranal also promoted neuron survival, stimulated neurogenesis, induced angiogenesis and increased SIRT1 expression *in vivo* and *in vitro*. Silencing of SIRT1 reversed the above effects of safranal on OGD/R-induced RBMEC. The present study indicated that safranal was a promising compound to exert neurorestorative effect in IS via upregulating SIRT1 expression. These results offer insight into developing new mechanisms in the recovery of neural function after safranal treatment of IS.

## Introduction

Cerebral ischemic stroke (IS) is a common cerebrovascular disease in clinic (1). It is a major cause of disability and the third leading cause of death after cancer and myocardial infarction worldwide (2,3). Generally, there are two clinical manifestations of stroke, IS and hemorrhagic stroke, and IS accounts for ~87% of all cases (4,5). As the only clinical medication approved by the US Food and Drug Administration, tPA must be administered within 4.5 h at the latest and therefore only few IS patients benefit from this drug (6,7). Researches have reported that angiogenesis and newborn neurons are crucial for the repair of cerebral ischemia and therefore are acknowledged as a promising therapeutic strategy for IS (8,9). Therefore, searching for a drug that facilitates angiogenesis and the survival and function of neurons is now the focus of basic research for IS treatment.

Saffron, as known as *Crocus sativus L*., is a plant belonging to Iridaceae family. It is widely cultivated in a great number of countries in the world, such as Turkey, Greece, India, Iran and China (10,11). In ancient times, saffron was used as the ingredient of energizing drink and orange jam (12). Nowadays, more and more pharmacological effects of saffron have been revealed, such as antioxidant, anti-tumor, anti-depressant, anticonvulsant, memory and learning enhancer and blood pressure regulator (13,14). More importantly, some experimental studies have demonstrated that saffron by oral administration can suppress neuronal death and lead to a decrease of infarct size in stroke rats (15,16). Safranal, a monoterpene aldehyde, is an active ingredient of saffron and responsible for the characteristic odor and aroma of this plant (17). The properties of safranal have been reported to include anti-inflammatory (18), anti-oxidant (19), antidiabetic (20), anti-carcinogenic (21) and anti-hypertensive (22) effects. Its neuroprotective role in neurological disorders has also been widely described, in Alzheimer's disease (23), epilepsy (24), spinal cord injury (25) and Parkinson's disease (26). Additionally, the protective role of safranal in the onset and development of IS has also been reported. For example, both Sadeghnia *et al* (27) and Hosseinzadeh *et al* (28) constructed a middle cerebral artery occlusion/reperfusion (MCAO/R) model in rats followed by administration with safranal via intraperitoneal injection. They concluded that the hippocampal cell loss and infarct volume of IS rats are markedly improved following safranal treatment and these improvements are due to the antioxidant activity of safranal (27,28). Forouzanfar *et al* (29) established an oxygen-glucose deprivation/reoxygenation (OGD/R) model in PC12 cells and indicated that safranal dramatically attenuates oxidative damage and suppresses apoptosis in OGD/R-induced PC cells. However, the detailed mechanism and the effects of safranal on angiogenesis and the repair of nerve system are remain to be elucidated.

The present study not only constructed a MCAO/R rat as *in vivo* model but also used OGD/R-induced rat brain microvascular endothelial cells (RBMEC) as *in vitro* model. The aim of the current study was to preliminarily ascertain the neurorestorative effects of safranal against IS and provide a theoretical basis the clinical therapy of IS patients.

## Materials and methods

### Reagents and cells

RBMEC and DMEM-H media containing 10% FBS were procured from BeNa Culture Collection. MilliporeSigma provided safranal (purity ≥90%). Bovine serum albumin (BSA) was purchased from Beijing Solarbio Science & Technology Co., Ltd. Terminal deoxynucleotidyl transferase dUTP nick end labeling (TUNEL) Apoptosis Detection Kit was obtained from AtaGenix and 2,3,5-triphenyltetrazolium chloride (TTC) solution (2%) was procured from Shanghai Macklin Biochemical Co., Ltd. Nissl staining solution, DAPI, Rat TGF-β1 enzyme-linked immunosorbent assay (ELISA) kit (cat. no. PT878), BCA protein assay kit, ECL detection kit, RIPA lysis buffer and Triton X-100 were from Beyotime Institute of Biotechnology. Rat VEGF ELISA kit (cat. no. ED-30908), Rat basic fibroblast growth factor 2 (bFGF-2) ELISA kit (cat. no. LCSCM30506) and Rat platelet-derived growth factor (PDGF) ELISA kit (cat. no. ED-34434) were obtained from Xiamen Lun Changshuo Biological Technology Co., Ltd. For western blotting, primary antibodies against silent information regulator 1 (SIRT1), brain-derived neurotrophic factor (BDNF), synaptophysin (SYN), microtubule associated protein 2 (MAP-2), GAPDH and HRP-conjugated secondary antibodies were from Proteintech Group, Inc.; nerve growth factor (NGF), neurotrophin-4 (NT-4) and Tau-1 were from Abcam; postsynaptic density protein 95 (PSD95) was from Cell Signaling Technology, Inc. For immunofluorescence staining, primary antibodies ionized calcium-binding adapter molecule 1 (Iba-1; cat. no. 66827-1-Ig) and tyrosine hydroxylase (TH; cat. no. 66334-1-Ig) were obtained from Proteintech Group, while primary antibody syntaxin (cat. no. ab188583) and fluorescein isothiocyanate-conjugated secondary antibody (cat. no. ab6717) were from Abcam. RNA extraction agent TRIzol^®^ reagent was obtained from Thermo Fisher Scientific, Inc. Hifair^®^ II 1st Strand cDNA Synthesis SuperMix and Hieff^®^ qPCR SYBR Green Master Mix were purchased from Shanghai Yeasen Biotechnology Co., Ltd. Small interfering (siRNA SIRT1-1/-2/-3 and the negative control (siRNA NC) were synthesized by Weizhen Biosciences. Transfection reagent Lipofectamine^®^ 3000 was obtained from Invitrogen (Thermo Fisher Scientific, Inc.). Dojindo Molecular Technologies, Inc. provided CCK-8 solution used for the measurement of cell viability. Matrigel obtained from BD Biosciences was used for tube formation assay.

### Establishment for MCAO/R rat model and neurological evaluation

A total of 30 healthy male Sprague-Dawley rats (280±20 g; 10 weeks old) were purchased from Vital River Laboratory Animal Technology (Pinghu, China) and housed in specific pathogen-free environments with a temperature of 22±2˚C and a humidity of 50-60%, under a 12 h light-dark cycle, with food and water available *ad libitum*. Following acclimatization in laboratory, rats were randomly divided into five groups: The sham, model, safranal (10 mg/kg), safranal (20 mg/kg) and safranal (40 mg/kg) groups, with six rats in each group. The MCAO/R rat model was established as previously described (30). Briefly, rats in each group were anesthetized using isoflurane (2% for induction and 1.5% for maintenance, in 80% N_2_O and 20% O_2_). MCAO/R was performed as follows: A midline incision was made to expose the right internal carotid artery and then a 6-0 nylon monofilament suture was inserted into the right internal carotid artery of each rat for artery occlusion for 2 h. Subsequently, the filament was withdrawn carefully to restore blood reperfusion. Rats in the sham group underwent the same surgical procedure apart from MCAO. Different doses of safranal (10, 20 and 40 mg/kg) were used to treat the model rats respectively via intraperitoneal injection for 28 consecutive days. Then, Zea-Longa and Ludmila Belayev scores were used to assess the neurological functions of rats, in a double-blind manner (31). The present study was conducted after obtaining the approval of Laboratory Animal Ethics Committee of Wenzhou Seventh People's Hospital (Wenzhou, China; approval no. 19JCYBJC00323) and the experimental procedures in rats were executed in compliance with the NIH Guide for the Care and Use of Laboratory Animals (8th edition; https://www.ncbi.nlm.nih.gov/books/NBK54050/).

### TTC staining

The rats were euthanized by exposure to 4% isoflurane followed by cervical dislocation, and death was confirmed by a lack of response to tail clamping. The brain tissues were harvested and cut into 2-mm-thick slices. The slices were stained with TTC solution (2%) at 37˚C for 15 min in the dark and then images were captured. Red represented the normal brain tissues, while pale color represented the ischemic brain tissues. The infarct size was assessed using ImageJ software (version 1.8; National Institutes of Health).

### Nissl staining

Brain paraffin sections were routinely dewaxed at room temperature with xylene I and xylene II, for 10 min each, and then gradient alcohol dehydration (100, 100, 95, 90, 80, 70 and 50%, each 5 min) was performed. Subsequently, the sections were stained with Nissl staining solution for 40 min at 50-60˚C. Sections were washed with deionized water, differentiated with Nissl differentiation solution, dehydrated with anhydrous ethanol, cleared with xylene and sealed with neutral gum. The stained hippocampal neurons were observed under a light microscope (Olympus Corporation; magnification, x400) and five areas were randomly selected from each group for statistical analysis.

### Immunofluorescence staining

Brain slices were deparaffinized with xylene (changed twice, 10 min each) and rehydrated in a graded ethanol series. After rinsing in PBS three times, the sections were underwent antigen retrieval in 10 mM Citrate buffer for 20 min at 95˚C. Following washing by PBS for three times, the slices were then permeabilized with 0.5% TritonX-100 in PBS at room temperature for 1 h. The sections were blocked with 5% BSA at room temperature for 1 h and were then incubated with primary antibodies Iba-1 (1:200; a marker of microglia), syntaxin (1:200; a marker of α-synuclein) and TH (1:200; a marker of dopaminergic neuron) overnight at 4˚C and then with the corresponding secondary antibodies labeled with fluorescent dyes (1:200) at 37˚C for 30 min. Following staining with DAPI at 37˚C for 15 min, images were captured using a fluorescence microscope (Olympus Corporation; magnification, x400).

### TUNEL assay

In accordance with the experimental operations in the manufacturer's specifications of the kit, the apoptosis was evaluated in brain tissues and RBMEC. The images were captured using a fluorescence microscope (Olympus Corporation; magnification, x400).

### ELISA

According to the instructions, the concentrations of VEGF, TGF-β1, bFGF-2 and PDGF in the serum and cortex of rats were determined via the corresponding commercial kits.

### Cell culture, OGD/R model establishment and safranal treatment

DMEM-H containing 10% FBS was used for the cultivation of RBMEC. The cells were maintained in a 5% CO_2_ and 95% air incubator at 37˚C. As previously reported (30), an *in vitro* OGD/R model was established with slight modification. In brief, RBMEC were cultured in a glucose-free DMEM-H medium for 6 h at 37˚C in a gas mix of 95% N_2_ and 5% CO_2_ for simulation of cerebral ischemia. Then, RBMEC were transferred into a normoxic incubator (95% air and 5% CO_2_) and incubated for 24 h to mimic reperfusion. RBMEC in the control group were cultured in normoxic environment and normal medium. In safranal-treated group, RBMEC subjected to ODG/R were treated with 50 µg/ml safranal for 12 h as previously described (32).

### Cell transfection

Using Lipofectamine^®^ 3000 (Invitrogen; Thermo Fisher Scientific, Inc.) RBMEC were co-transfected with siRNA SIRT1-1 (5'-CAGTTTCATAGAGCCATGAAGTATG-3'), siRNA SIRT1-2 (5'-CCAGTAGCACTAATTCCAAGTTCTA-3'), siRNA SIRT1-3 (5'-GCTACACTTGTAGACCAAACAATAA-3') or siRNA NC (5'-GCTTTCAGATGACCAACAAACATAA-3') for 48 h at 37˚C. The concentration of all siRNA agents was 50 nM. After that, 48 h after transfection, RBMEC were gathered and the gene expression levels of SIRT1 were detected through reverse transcription-quantitative (RT-q) PCR.

### Total RNA isolation and RT-qPCR

According to the manufacturer's protocols, total RNA extraction from brain cortex and RBMEC (2x10^5^) was implemented using TRIzol^®^, followed by synthesizing cDNA products with the aid of Hifair II 1st Strand cDNA Synthesis SuperMix. Following the instructions of manufacturer, RT-qPCR analysis was carried out using the Hieff qPCR SYBR Green Master Mix. The following thermocycling conditions were used for the qPCR: Initial denaturation at 95˚C for 3 min, followed by 40 cycles at 95˚C for 15 sec, annealing at 60˚C for 30 sec, elongation at 72˚C for 1 min and a final extension at 72˚C for 5 min. GAPDH was used for normalization. The mRNA expression levels were calculated by applying the 2^-ΔΔCq^ approach (33). The primers used are listed in Table I.

### Western blotting

Cortex tissues or RBMEC were lysed with RIPA lysis buffer on ice for 15 min. A BCA protein assay kit was used for examining total protein content. After separating the protein products (50 µg protein/lane) on 10% gels using SDS polyacrylamide gel electrophoresis, they were transferred onto PVDF membranes, blocked with 5% nonfat milk for 2 h at 25˚C. Subsequently, primary antibodies including SIRT1 (1:20,000), BDNF (1:2,000), SYN (1:20,000), MAP-2 (1:20,000), NGF (1:3,000), NT-4 (1:3,000) and Tau-1 (1:1,000), PSD95 (1:1,000) and GAPDH (1:50,000) were added to the membranes for incubation overnight at 4˚C. Then, the membrane was placed in HRP-conjugated secondary antibodies (1:1,000), along with incubation for 1 h at room temperature. The protein bands were developed with an ECL detection Kit and analyzed with fully automatic chemiluminescence imaging system (Tanon 5200Multi; Tanon Science & Technology).

### CCK-8 assay

RBMEC (5,000 cells/well) was plated onto a 96-well plate. CCK-8 solution (10 µl) was mixed with the medium. Following 2 h of incubation, RBMEC were exposed to 10 µl stop buffer. Cell viability was evaluated by examining the absorbance at 450 nm.

### Wound healing assay

RBMEC were seeded in 6-well plates with a density of 5x10^4^ cells/well. When the cells had grown to 100% confluence, a scratch was conducted in the monolayer cells using a pipette tip (10 µl) and RBMEC were then incubated in a serum-free DMEM-H medium for 48 h. The width of scratches at 0 h or 48 h was recorded under a microscope. The migrative potentials of RBMEC were analyzed using an ImageTool software (UTHSCSA) with the following formula: (the width at 0 h-the width at 48 h)/the width at 0 h x100.

### Tube formation experiments

RBMEC (5,000 cells/well) were plated into a 24-well plate that pre-coated with Matrigel at 37˚C for 30 min, followed by incubation in DMEM-H supplemented with 10% FBS at 37˚C for 24 h. Tube formation was observed under a microscope. Tube length and branch were analyzed using an ImageJ software (version 1.8; National Institutes of Health).

### Statistical analysis

*In vitro* experiments were performed in triplicate, and each experiment was repeated three times. *In vivo* experiments were performed using 6 rats per group. The variations among the data were evaluated by one-way ANOVA, followed by Tukey's multiple comparison test. Data analysis was performed with SPSS software v22.0 (IBM Corp.). Measurement data were displayed as the mean ± standard deviation. P<0.05 was considered to indicate a statistically significant difference.

## Results

### Safranal ameliorates neuronal injury in a MCAO/R rat model

**Figure 1:**
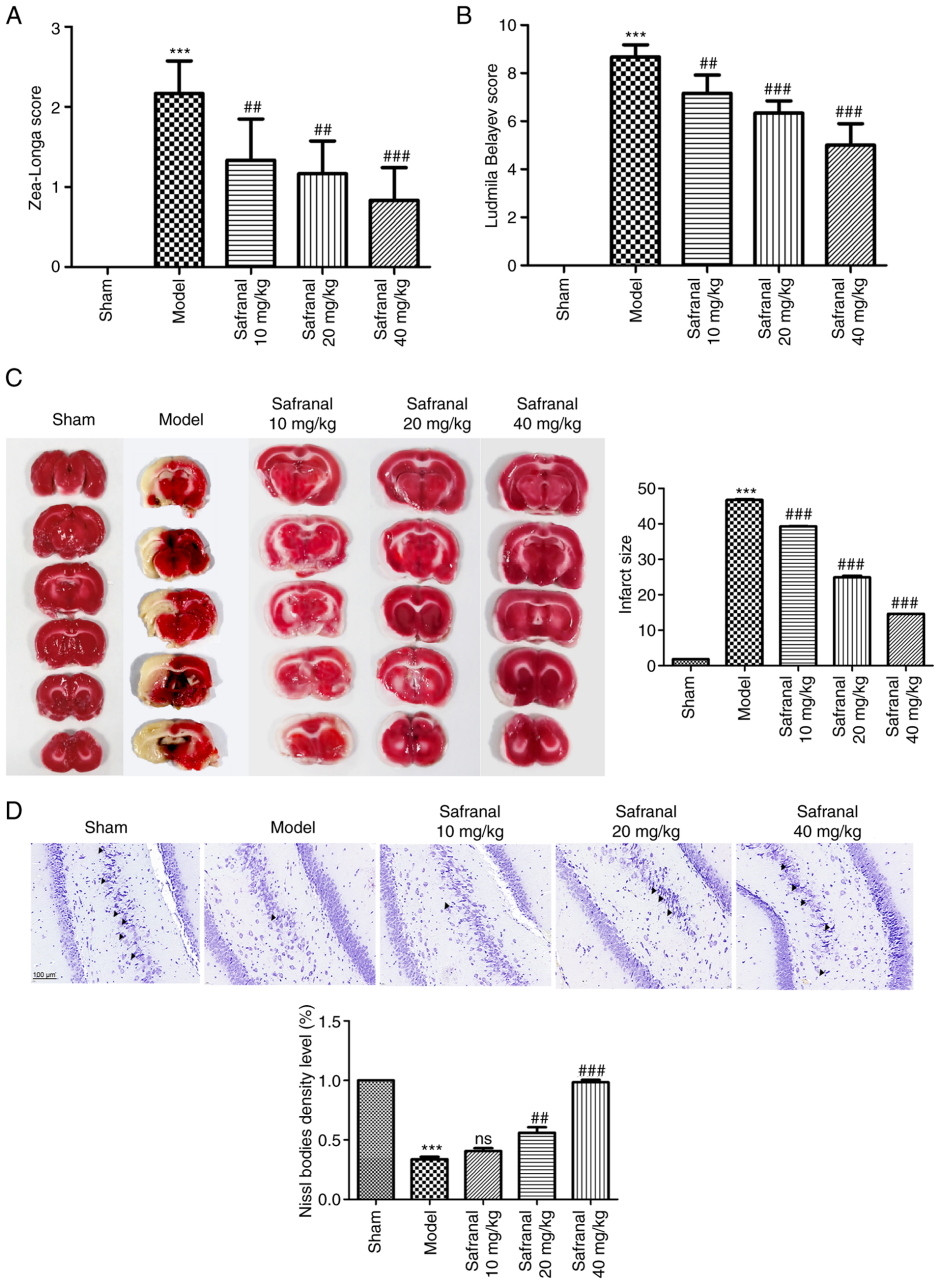
Safranal ameliorates neuronal injury in a MCAO/R rat model. Neurological function assessment for each group using (A) Zea-Longa and (B) Ludmila Belayev scores. (C) Infarct size in each group was calculated by TTC staining. (D) Nissl staining showed the pathological changes in brain tissues; magnification, x400. ^***^P<0.001 vs. sham. ^##^P<0.01, ^###^P<0.001 vs. model. *In vivo* experiments were performed using six rats per group. MCAO/R, middle cerebral artery occlusion/reperfusion; TTC, 2,3,5-triphenyltetrazolium chloride.

Zea-Longa 5-point method and Ludmila Belayev 12-point method were used to assess the neurological functions of MCAO/R models. As shown in Fig. 1A and B, compared with the sham group, the Zea-Longa score and Ludmila Belayev score in the model group were markedly increased (P<0.001). However, the neurological functions of rats in the MCAO/R models were notably improved following safranal treatment (P<0.01). The cerebral infarct size was then evaluated via TTC staining. A larger infarct size was observed in the model group as compared with the sham group (Fig. 1C; P<0.001). Safranal treatment could reduce infarct size remarkably, especially for 40 mg/kg of safranal (P<0.001). As illustrated in Fig. 1D, Nissl staining showed that the neurons in the sham group were arranged neatly and densely, with abundant Nissl bodies. The arrangement of neurons in the model group was loose, with a large number of vacuolar structures and a significant decrease of Nissl bodies. In contrast to the model group, the loss of Nissl bodies was significantly improved and the activity of neuron was enhanced in safranal treatment group. Overall, high dose of safranal (40 mg/kg) seemed to decrease the Zea-Longa score and Ludmila Belayev score, reduce infarct size and improve the loss of Nissl bodies more effectively compared with lower doses. These results implied that high dose of safranal (40 mg/kg) was more helpful for ameliorating neuronal injury in a MCAO/R rat model.

### Safranal represses microglia over-activation, synuclein aggregation and dopaminergic neurodegeneration

**Figure 2:**
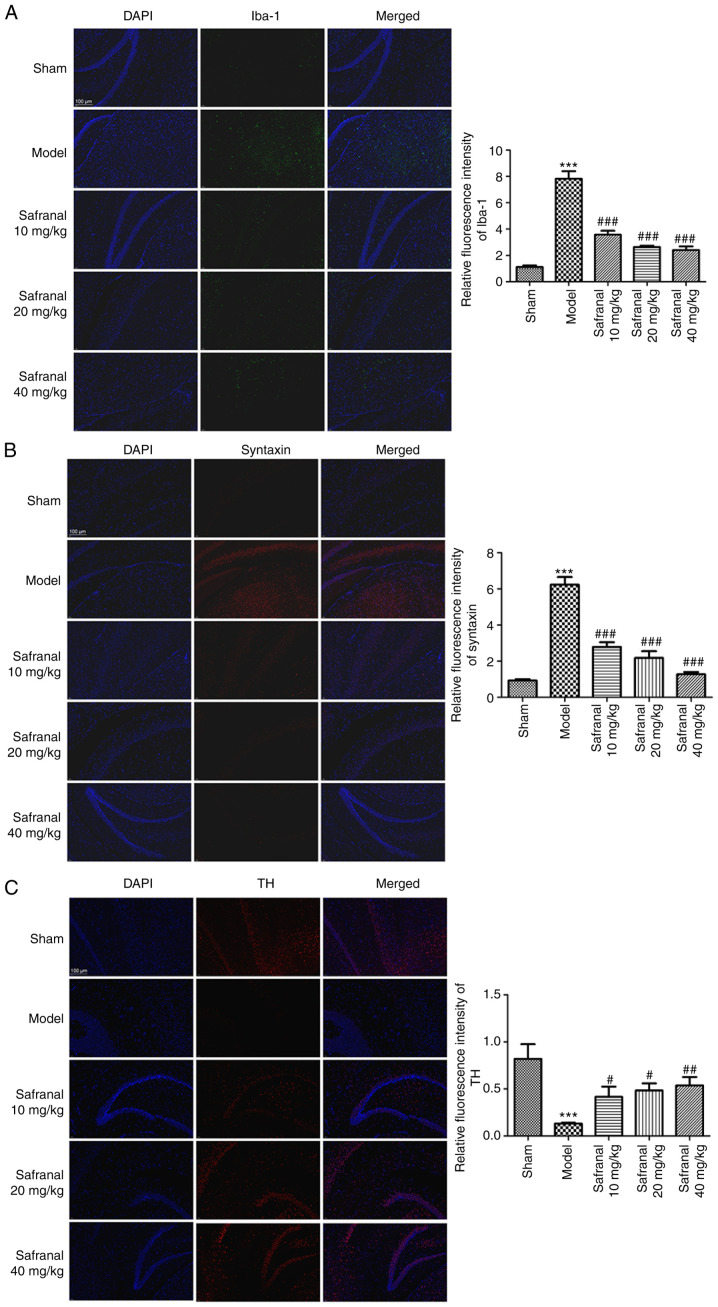
Safranal represses microglia over-activation, synuclein aggregation and dopaminergic neurodegeneration. The relative fluorescence intensities of (A) Iba-1, (B) syntaxin and (C) TH in different groups were calculated by immunofluorescence; magnification, x400. ^***^P<0.001 vs. sham. ^#^P<0.05, ^##^P<0.01, ^###^P<0.001 vs. model. *In vivo* experiments were performed using six rats per group. Iba-1, ionized calcium-binding adapter molecule 1; TH, tyrosine hydroxylase.

Microglia over-activation, synuclein aggregation and dopaminergic neuron loss are considered important pathological features in neuronal injury-related diseases including, IS (34,35). As shown in Fig. 2A and B, the results of immunofluorescence demonstrated that the relative fluorescence intensity of Iba-1 and synuclein in the model group was markedly increased compared with that of the sham group (P<0.001). However, in different safranal treatment groups, the relative fluorescence intensity of Iba-1 and synuclein was significantly weaker (P<0.001). The opposite results were observed in the relative fluorescence intensity of TH (Fig. 2C; P<0.05). Additionally, it was found that 40 mg/kg of safranal seemed to have relatively stronger suppressive effect on the levels of Iba-1 and synuclein, and relatively stronger alleviative effect on TH level. These data suggested that high dose of safranal (40 mg/kg) can significantly repress microglia over-activation, synuclein aggregation and dopaminergic neurodegeneration.

### Safranal suppresses neuron apoptosis and promotes angiogenesis in a MCAO/R rat model

**Figure 3:**
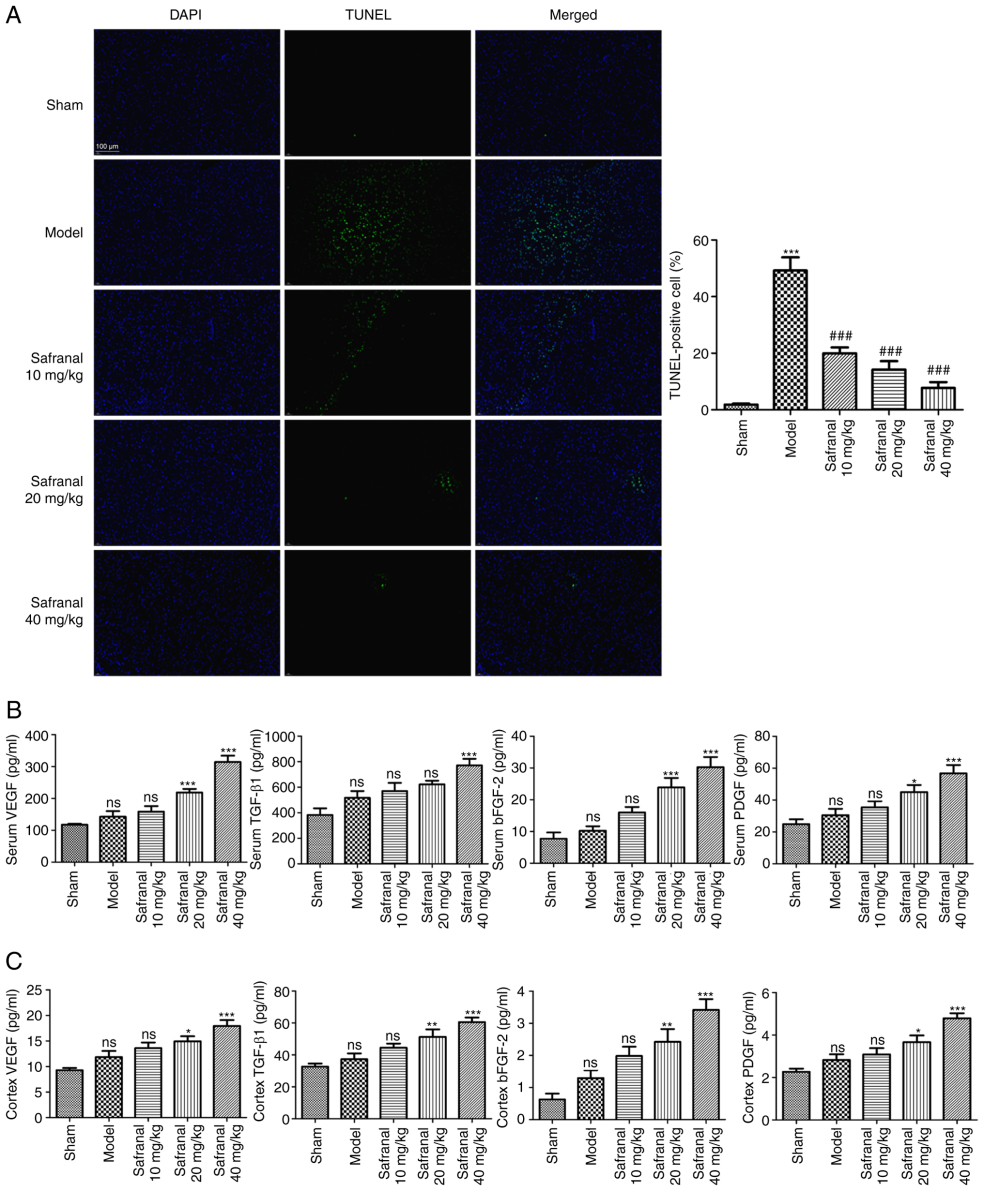
Safranal suppresses neuron apoptosis and promotes angiogenesis in a MCAO/R rat model. (A) TUNEL-positive cells in different groups were determined by TUNEL assay; magnification x400. ^***^P<0.001 vs. sham. ^###^P<0.001 vs. model. (B) The concentrations of VEGF, TGF-β1, bFGF-2 and PDGF in the serum of rats were measured by ELISA. (C) The concentrations of VEGF, TGF-β1, bFGF-2 and PDGF in the cortex of rats were measured by ELISA. ^*^P<0.05, ^**^P<0.01, ^***^P<0.001 vs. model; ns, no significance. *In vivo* experiments were performed using six rats per group. MCAO/R, middle cerebral artery occlusion/reperfusion; TUNEL, terminal deoxynucleotidyl transferase dUTP nick end labeling; bFGF-2, basic fibroblast growth factor 2; PDGF, platelet-derived growth factor; ELISA, enzyme-linked immunosorbent assay.

As the survival of neurons in brain tissues is neuroprotective in IS, the effect of safranal on neuron apoptosis was determined via TUNEL assay. TUNEL-positive cells in the model group were significantly increased, whereas were clearly reduced following safranal treatment (Fig. 3A; P<0.001). Angiogenic factors play an important role in neovascularization after nerve injury. Safranal treatment, especially for 40 mg/kg of safranal, markedly increased the levels of VEGF, TGF-β1, bFGF-2 and PDGF in the serum and cortex of MCAO/R rats (Fig. 3B and C; P<0.05). The above results indicated that high dose of safranal (40 mg/kg) can suppress neuron apoptosis and promotes angiogenesis effectively in a MCAO/R rat model.

### Safranal repairs nervous system through upregulating SIRT1 expression

**Figure 4:**
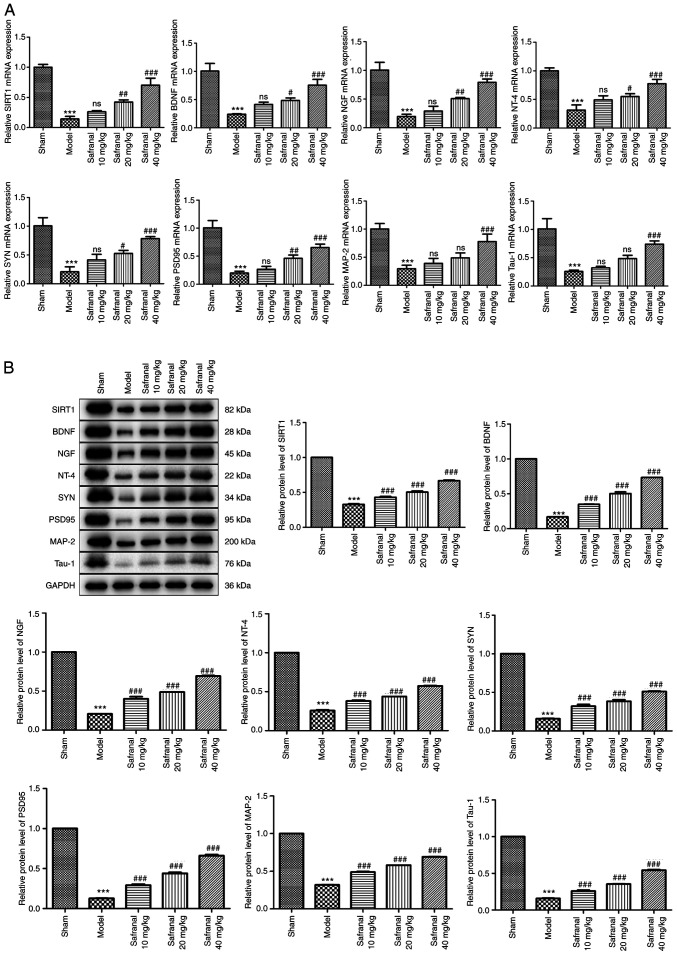
Safranal repairs nervous system through upregulating SIRT1 expression. (A) The mRNA expression of SIRT1, BDNF, NGF, NT-4, SYN, PSD95, MAP-2 and Tau-1 in different groups was measured by reverse transcription-quantitative PCR. (B) The protein levels of SIRT1, BDNF, NGF, NT-4, SYN, PSD95, MAP-2 and Tau-1 in different groups were determined by western blotting. ^***^P<0.001 vs. sham. ^#^P<0.05, ^##^P<0.01, ^###^P<0.001 vs. model; ns, no significance. *In vivo* experiments were performed using six rats per group. SIRT1, silent information regulator 1; BDNF, brain-derived neurotrophic factor; NGF, nerve growth factor; NT-4, neurotrophin-4; SYN, synaptophysin; PSD95, postsynaptic density protein 95; MAP-2, microtubule associated protein 2; NGF, nerve growth factor; NT-4, neurotrophin-4.

Activation of SIRT1 has been confirmed to exert neuroprotection against cerebral IS (36). Neurotrophins such as BDNF, NGF and NT-4 have been demonstrated to promote neurogenesis after cerebral ischemia (37). Additionally, a previous study demonstrated that dendritic remodeling and synaptogenesis are important for neural restoration (38). The present study therefore explored the effects of safranal treatment on the restoration of neural function. As shown in Fig. 4A, the mRNA expression of SIRT1, BDNF, NGF, NT-4, SYN, PSD95, MAP-2 (a somato-dendritic marker) and Tau-1 (an axonal marker) was decreased in the model group in contrast to the sham group (P<0.001). High dose of safranal (40 mg/kg) significantly increased the mRNA expression of SIRT1, BDNF, NGF, NT-4, SYN, PSD95, MAP-2 and Tau-1 (P<0.001). Similarly, the protein levels of these factors were also markedly elevated following safranal treatment (Fig. 4B; P<0.001). These results implied that high dose of safranal (40 mg/kg) can repair nervous system by upregulating SIRT1 expression.

### Silencing of SIRT1 reverses the effects of safranal on cell growth, migration, apoptosis and tube formation in OGD/R-induced RBMEC

**Figure 5:**
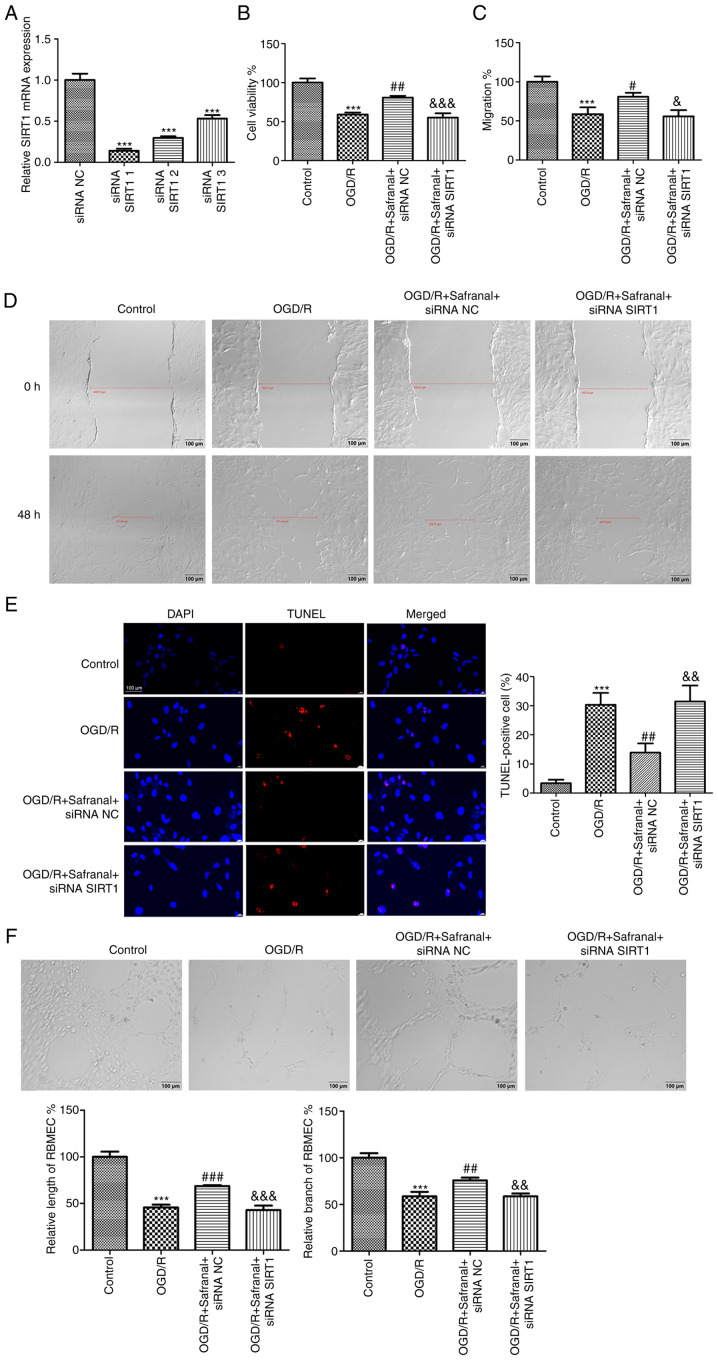
Silencing of SIRT1 reverses the effects of safranal on cell growth, migration, apoptosis and tube formation in OGD/R-induced RBMEC. (A) The mRNA expression of SIRT1 in RBMEC transfected with siRNA NC or siRNA SIRT1-1/-2/-3 was measured by reverse transcription-quantitative PCR. ^***^P<0.001 vs. siRNA NC. (B) The viability of OGD/R-induced RBMEC in different groups was calculated via CCK-8 assay. (C and D) The migrative potentials of OGD/R-induced RBMEC were assessed through wound healing assay; scale bar, 100 µm. (E) TUNEL-positive cells in different groups were determined by TUNEL assay; magnification x 400. (F) Tube formation assay in OGD/R-induced RBMEC; scale bar, 100 µm. ^***^P<0.001 vs. control. ^#^P<0.05, ^##^P<0.01, ^###^P<0.001 vs. OGD/R. ^&^P<0.05, ^&&^P<0.01, ^&&&^P<0.001 vs. OGD/R + safranal + siRNA NC. *In vitro* experiments were performed in triplicate, and each experiment was repeated three times. SIRT1, silent information regulator 1; OGD/R, oxygen-glucose deprivation/reoxygenation; RBMEC, rat brain microvascular endothelial cells; si, short interfering; NC, negative control; TUNEL, terminal deoxynucleotidyl transferase dUTP nick end labeling.

The interaction of safranal with SIRT1 was ascertained in OGD/R-induced RBMEC. First, siRNA SIRT1-1/-2/-3 or siRNA NC was introduced into RBMEC. As shown in Fig. 5A, SIRT1 had the lowest expression level in RBMEC when transfected with siRNA SIRT1-1 (P<0.001). Therefore, siRNA SIRT1-1 was selected for the succeeding tests. CCK-8 and wound healing assays demonstrated that the viability and migrative potential of RBMEC could be suppressed by OGD/R challenge (Fig. 5B-D; P<0.001), but were markedly enhanced following safranal treatment (P<0.05). Notably, transfection of siRNA SIRT1 notably reversed the promoting effects of safranal on the viability and migration of OGD/R-induced RBMEC (P<0.05). For the effect of safranal interaction with SIRT1 on the apoptosis of OGD/R-induced RBMEC, a large number of TUNEL-positive cells were found in the OGD/R group as compared with the control group (Fig. 5E; P<0.001). In the OGD/R + safranal + siRNA NC group, the number of TUNEL-positive cells was significantly reduced (P<0.01). Moreover, knocking down SIRT1 attenuated the repressive effect of safranal on the number of TUNEL-positive cells (P<0.01). The angiogenesis of RBMEC was detected by tube formation assay. It was demonstrated that safranal treatment promoted the tube formation of RBMEC, with visual representation of increased total tube length and branches (Fig. 5F; P<0.01) and at the same time, siRNA SIRT1 transfection in OGD/R-induced RBMEC in presence of safranal suppressed total tube and branch length (P<0.01).

### Effects of interaction of safranal with SIRT1 on the levels of neurotrophins and synaptogenesis-related factors

**Figure 6:**
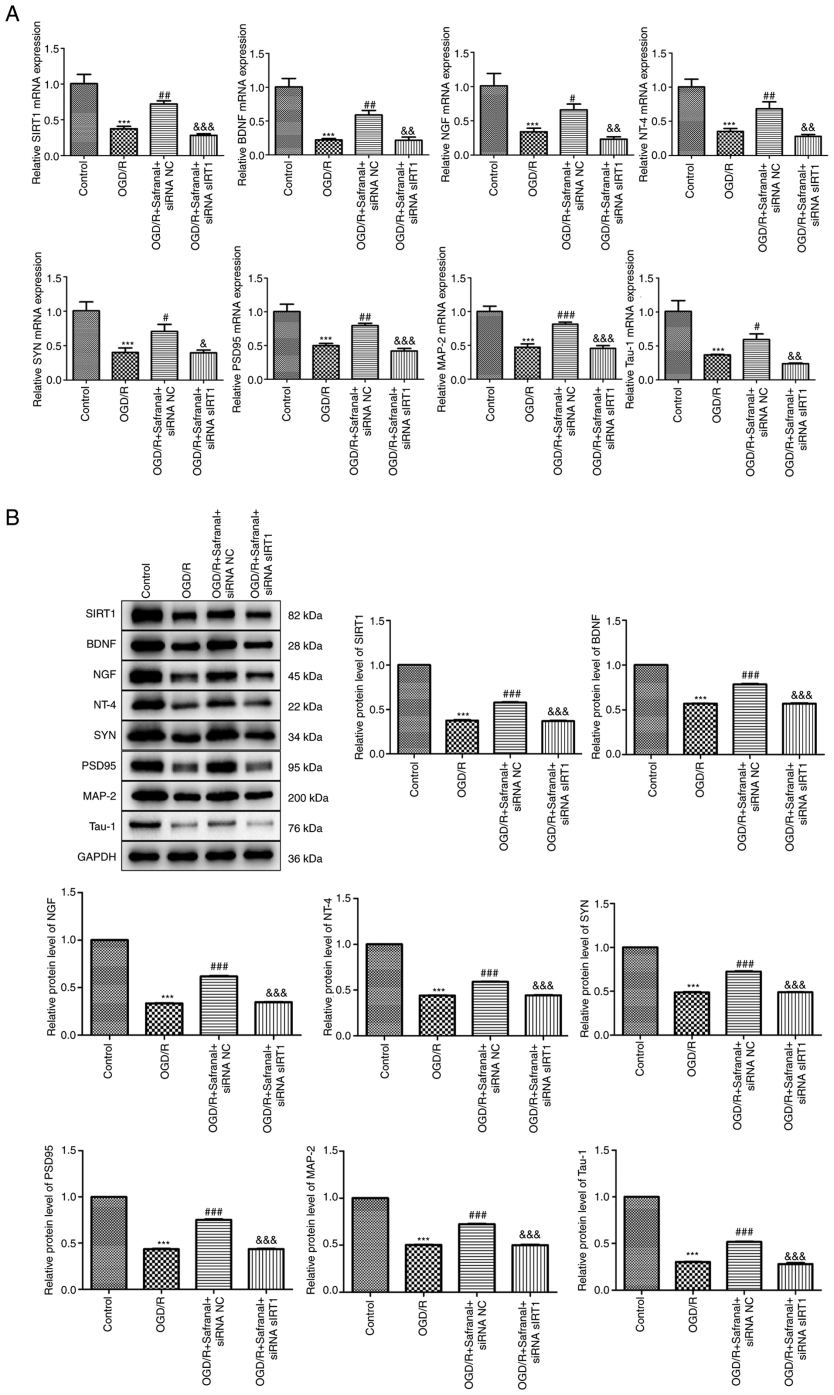
Effects of interaction of safranal with SIRT1 on the levels of neurotrophins and synaptogenesis-related factors. (A) The mRNA expression of SIRT1, BDNF, NGF, NT-4, SYN, PSD95, MAP-2 and Tau-1 in OGD/R-induced RBMEC was measured by RT-qPCR. (B) The protein levels of SIRT1, BDNF, NGF, NT-4, SYN, PSD95, MAP-2 and Tau-1 in OGD/R-induced RBMEC were determined by western blotting. ^***^P<0.001 vs. control. ^#^P<0.05, ^##^P<0.01, ^###^P<0.001 vs. OGD/R. ^&^P<0.05, ^&&^P<0.01, ^&&&^P<0.001 vs. OGD/R + safranal + siRNA NC. *In vitro* experiments were performed in triplicate, and each experiment was repeated three times. SIRT1, silent information regulator 1; BDNF, brain-derived neurotrophic factor; NGF, nerve growth factor; NT-4, neurotrophin-4; SYN, synaptophysin; PSD95, postsynaptic density protein 95; MAP-2, microtubule associated protein 2 OGD/R, oxygen-glucose deprivation/reoxygenation; RBMEC, rat brain microvascular endothelial cells; si, short interfering; NC, negative control.

As the effects of safranal on the restoration of neural function had been ascertained in MCAO/R rat model as described earlier, the effects of interaction of safranal with SIRT1 on the levels of neurotrophins and synaptogenesis-related factors in OGD/R-induced RBMEC needed to be further validated. As shown in Fig. 6A and B, the mRNA expression and protein levels of SIRT1, BDNF, NGF, NT-4, SYN, PSD95, MAP-2 and Tau-1 in the OGD/R group were markedly decreased relative to those in the control group (P<0.001). As expected, the mRNA expression and protein levels of these factors were restored to some extent in the OGD/R + safranal + siRNA NC group (P<0.05), but were further reduced in OGD/R + safranal + siRNA SIRT1 group (P<0.05).

## Discussion

IS is a serious medical problem globally and previous researches have reported that there are 15 million patients suffered from IS every year, with ~6.5 million mortalities and ~1/3 of patients suffered permanent disability due to neuronal damage (39-41). Therefore, IS has become an enormous burden to the healthcare system worldwide. Although tPA is currently the best available option for IS patients, the short therapeutic window and some adverse effects such as intracranial hemorrhage have limited its clinical application (42). In the ischemic penumbra, neuron death and low cerebrovascular density are the main features of IS patients (43,44). However, little success in the therapeutics that promote neural repair due to the complex mechanisms of IS process (45). The current study identified the neurorestorative role and mechanism of safranal in IS, indicating that safranal can promoting neuron survival, angiogenesis and neurogenesis to attenuate IS progression via regulating SIRT1 expression.

MCAO/R is the most frequently used method to mimic IS in murine models. In the present study, based on the experimental results that rats in the model group exhibited significantly higher neurological scores, Nissl body loss and increased infarct size, a MCAO/R rat model was successfully established. Additionally, it was found that high dose of safranal (40 mg/kg) can significantly attenuate these symptoms, which was in line with the data from Sadeghnia *et al* (27) indicating that safranal has protective effects on MCAO/R rats. A previous study indicated that angiogenesis is conducive to the recovery of blood supply in ischemic penumbra, thus promoting the activation of neurons (46). Angiogenesis is a process that enhances the proliferative and migrative potentials of vascular endothelial cells and the reconstruction of new vascular networks (47). It is a complex process regulated by pro-angiogenesis and anti-angiogenesis cytokines. VEGF is considered as the most indispensable cytokine to promote angiogenesis (43). TGF-β1 and bFGF-2 are both pro-angiogenesis and are involved in the migration and proliferation of endothelial cells (46). PDGF combined with its receptor are responsible for recruiting brain pericytes into newly formed vessels during the progression of angiogenesis (48). Therefore, it was hypothesized that safranal may exert a neuroprotective role by inhibiting endothelial cell apoptosis and promoting angiogenesis. As expected, it was found that safranal treatment, especially at 40 mg/kg, markedly elevated the levels of VEGF, TGF-β1, bFGF-2 and PDGF in both serum and cortex. In addition, it also suppressed the percentage of TUNEL-positive cell to a great extent. These results corroborated the hypothesis.

Furthermore, the over-activation of microglia, α-synuclein aggregation and dopaminergic neurodegeneration are considered as the important pathological features in neuronal injury-related diseases including IS (34,35). A previous study revealed that safranal can drive the inhibition and disaggregation of synuclein fibrils (49). It was considered that safranal treatment may suppress microglia over-activation, synuclein aggregation and dopaminergic neuron loss in MCAO/R rats. The results that high dose of safranal (40 mg/kg) distinctly attenuated the fluorescence intensities of Iba-1 and syntaxin and enhanced TH fluorescence intensity validated this hypothesis. Dendrites and axons are two main structures in mature neurons. It is reported that dendritic remodeling and synaptogenesis can promote the neurological activity in the cerebral cortex (38). Moreover, neurons can release neurotransmitters to regulate the populations of neurons (50). In the results of the present study, a high dose of safranal (40 mg/kg) not only significantly increased the levels of SYN and PSD95, but also elevated the levels of axon and dendrite markers Tau-1 and MAP-2. These results implied that safranal may be beneficial for the differentiation of neuron. In addition, neurotrophins such as BDNF, NGF and NT-4 have been reported to be related to neurogenesis (37). BDNF has been shown to reduce neuron death and promote neuroplasticity and neurite growth (41). NGF can stimulate the formation of axonal sprouting and induce axonal functional reconnection (51). NT-4 is involved in the differentiation and regeneration of neurons (52). The present study therefore further ascertained the effects of safranal on these neurotrophins. As expected, safranal at 40 mg/kg also clearly increased the levels of BDNF, NGF and NT-4. All the above results confirmed the neurorehabilitation role of safranal in IS development.

SIRT1 is a type of histone deacetylase belong to sirtuin family (53). An anatomical study conducted by Zakhary *et al* (54) indicated that SIRT1 is mainly distributed in basal ganglia, prefrontal cortex and hippocampus of the rodent and human nervous systems. A large number of researches has demonstrated that SIRT1 can be activated by some compounds to alleviate IS through several mechanisms, namely antioxidative, antiapoptotic and anti-inflammatory effects. For example, α-lipoic acid can alleviate the damage to the ischemic brain by reducing oxidative damage through activation of SIRT1(55). Salvianolic acid B treatment increases the expression of SIRT1 and then leads to a decrease of Bax and an increase of Bcl-2, finally attenuating IS development via inhibiting neuron apoptosis (56). Another study indicated that resveratrol acts as an activator of SIRT1 to exert neuroprotective effect in MCAO/R rats (57). Based on the protective role of safranal in MCAO/R rats obtained in the present study, it was hypothesized that safranal may promote angiogenesis and neurogenesis by upregulating SIRT1 expression. First, the present study found that safranal upregulated the expression of SIRT1 both *in vivo* and *in vitro*. This finding implied that SIRT1 was indeed a molecular target of safranal. The interaction between safranal and SIRT1 was then explored in OGD/R-induced RBMEC. The present study demonstrated that silencing of SIRT1 reversed the promoting effects of safranal on the viability and migration of OGD/R-induced RBMEC and the inhibiting effect on apoptosis. Knocking down SIRT1 also suppressed RBMEC tube formation caused by safranal. These results confirmed the antiapoptotic and angiogenesis roles of safranal in IS progression. Additionally, in OGD/R-induced RBMEC, it was further indicated that silencing of SIRT1 also reversed the enabling effects of safranal on the levels of BDNF, NGF, NT-4, SYN, PSD95, MAP-2 and Tau-1. It was considered that safranal can induce neurogenesis through upregulating SIRT1 expression.

Briefly, the present study mainly analyzed the neurorestorative role of safranal in MCAO/R rats and OGD/R-induced RBMEC and indicated that safranal can promote neuron survival, angiogenesis and neurogenesis to attenuate IS progression by upregulating SIRT1 expression. This may contribute more information and insights on the underlying action mechanism of safranal for IS treatment in clinical practice.

## Figures and Tables

**Table I:** Real-time PCR primer synthesis list.

Gene	Sequences
SIRT1	
Forward	5'-ATCTCCCAGATCCTCAAGCCA-3'
Reverse	5'-CTTCCACTGCACAGGCACAT-3'
BDNF	
Forward	5'-AATAATGTCTGACCCCAGTGCC-3'
Reverse	5'-ATTGTTGTCACGCTCCTGGT-3'
NGF	
Forward	5'-GAGCGCATCGCTCTCCTT-3'
Reverse	5'-GTGTGAGTCGTGGTGCAGTA-3'
NT-4	
Forward	5'-AGGACCCTGACTTACCCTGG-3'
Reverse	5'-CCTAGCCCCAGCTCATACAT-3'
SYN	
Forward	5'-TACAGCCGTGTTCGCTTTCA-3'
Reverse	5'-GTGGCCATCTTCACATCGGA-3'
PSD95	
Forward	5'-CCGCTACCAAGATGAAGACAC-3'
Reverse	5'-GTTCCATTCACCTGCAACTCA-3'
MAP-2	
Forward	5'-CTGCACTGGAAGAAGCCTCG-3'
Reverse	5'-GAGGAACTAAGGCAGCGTGT-3'
Tau-1	
Forward	5'-TCCTCGCCTCCTGTCGATTA-3'
Reverse	5'-AGCTTGGTCCTCCATGTTCG-3'
GAPDH	
Forward	5'-TCAAGAAGGTGGTGAAGCAGG-3'
Reverse	5'-TCAAAGGTGGAGGAGTGGGT-3'

## Data Availability

The datasets analyzed during the current study are available from the corresponding author on reasonable request.
